# Impact of COVID-19 on HIV services and anticipated benefits of vaccination in restoring HIV services in Ethiopia: A qualitative assessment

**DOI:** 10.3389/fpubh.2022.1033351

**Published:** 2022-11-03

**Authors:** Abebe Feyissa Amhare, Min Zhao, Janet Seeley, Wei Hong Zhang, Girma Garedew Goyomsa, Tinsae Abeya Geleta, Rui Zhao, Lei Zhang

**Affiliations:** ^1^China-Australia Joint Research Center for Infectious Diseases, School of Public Health, Xi'an Jiaotong University Health Science Center, Xi'an, China; ^2^College of Health Science, Salale University, Fiche, Ethiopia; ^3^Department of Public Health and Primary Care, Faculty of Medicine and Health Sciences, University of Ghent, Ghent, Belgium; ^4^Department of Global Health and Development, Faculty of Public Health and Policy, London School of Hygiene and Tropical Medicine, London, United Kingdom; ^5^Department of Public Health and Primary Care, Faculty of Medicine and Health Sciences, University of Ghent, Ghent, Belgium; ^6^School of Public Health, Université Libre de Bruxelles, Brussels, Belgium; ^7^School of Humanities and Management, Institute of Life Culture, Guangdong Medical University, Dongguan, China; ^8^Melbourne Sexual Health Centre, Alfred Health, Melbourne, VIC, Australia; ^9^Central Clinical School, Faculty of Medicine, Nursing and Health Sciences, Monash University, Melbourne, VIC, Australia

**Keywords:** COVID-19, people living with HIV, healthcare interruption, COVID-19 vaccine, Ethiopia

## Abstract

**Background:**

HIV services were inevitably disrupted and affected due to COVID-19. There are many challenges in implementing appropriate HIV services, particularly in the provision of health care and the link between people living with HIV/AIDS and retention in care. The study investigated the impact of COVID-19 on HIV services and the anticipated benefit of the COVID-19 vaccination on HIV service restoration in North Shewa, Oromia, Ethiopia.

**Methods:**

A qualitative descriptive study approach was used to explore how healthcare delivery evolved during the outbreak of COVID-19 in Ethiopia. Sixteen antiretroviral therapy (ART) clinics were selected from 13 districts and one administrative town in Ethiopia. From them, 32 ART providers were purposively selected based on their experience in ART provision. Data were collected from June to July 2021 using in-depth interviews. A thematic analysis approach was used to analyze the data, based on themes and subthemes emerging from the data. ATLAS.ti software was used for coding.

**Results:**

Healthcare for people living with HIV was interrupted due to the COVID-19 pandemic. Medical appointments, HIV testing and counseling services, opportunistic infection treatment, medicine supply, and routine viral load and CD_4_ T-cell count tests were interrupted. Due to a shortage of healthcare staff, outreach testing services and home index testing were discontinued and HIV testing was limited only to hospitals and health centers. This has substantially affected accessibility to HIV testing and reduced the quality of HIV service delivery. Telehealth and less frequent visits to health facilities were used as alternative ways of delivering HIV services. The COVID-19 vaccination campaign is expected to restore healthcare services. Vaccination may also increase the confidence of healthcare providers by changing their attitudes toward COVID-19.

**Conclusions:**

The COVID-19 pandemic has substantially impacted HIV services and reduced the quality of HIV care in Ethiopia. Health facilities could not provide routine HIV services as they prioritize the fight against COVID-19, leading to an increase in service discontinuation and poor adherence.

## Introduction

The outbreak of Coronavirus Disease 2019 (COVID-19) is a rapidly developing global public health crisis that has affected the lives of millions of people (1, 2). Since the beginning of the COVID-19 pandemic, most deaths have occurred in the elderly and/or in the presence of chronic diseases such as diabetes (DM), HIV, hypertension, obesity, chronic kidney disease, cardiovascular disease, and cancer (3). People living with HIV (PLHIV) are considered medically and socially vulnerable during the coronavirus [Severe Acute Respiratory Syndrome Coronavirus (SARS-CoV-2)] outbreak period (4). In particular, people with unsuppressed viral loads are at increased risk of a serious outcome from SARS-CoV2 infection (5, 6).

HIV/AIDS-related services are inevitably disrupted and affected due to COVID-19 (7–11). There are many challenges in implementing appropriate HIV services, particularly in the provision of health care and the link between people living with HIV/AIDS and care retention (7). People who are retained in care are more likely to adhere to antiretroviral therapy and experience improved health outcomes and are less likely to transmit HIV to others (12). Missed medical appointments are independently associated with an increased risk of AIDS and death (13, 14). Traditionally, retention in care has relied on the ability of people living with HIV to meet regularly with their HIV medical team, which in many places has been difficult to do during the COVID-19 pandemic. Treatment and prevention services for HIV have been severely interrupted (15–17).

In higher-income countries during the implementation of non-pharmacological prevention measures such as lockdowns and physical distancing during the COVID-19 outbreak telemedicine replaced face-to-face appointments with medical service providers (18, 19). To prevent PLHIV from COVID-19, telehealth is recommended to replace treatment at health facilities (20–25). Telehealth is defined as the provision and promotion of health and health-related services through telecommunications and digital communication technologies (26, 27). It can increase the survival rate of PLHIV during public health emergencies by implementing inclusive multi-level strategies for non-technologically disadvantaged populations living with HIV (28). In low- and middle-income countries such as Ethiopia, using telehealth has many barriers, not all patients have smart or even mobile phones and internet access.

Ethiopia confirmed its first case of COVID-19 on March 13, 2020 (29). Since then, many health facilities providing HIV prevention, treatment, and care services had to change their schedules and operation modalities to adapt to the challenges imposed by COVID-19 and its prevention and control measures (social distancing, travel restrictions, stay-at-home orders, etc.). Routine, non-urgent, or elective health care visits and procedures were canceled or delayed. Supportive services, such as face-to-face counseling, housing services, and outreach services, were temporarily suspended. In addition, patients avoided accessing health care, even for urgent concerns, due to fear of COVID-19 exposure (17, 30, 31).

Despite the high burden of COVID-19 in low- and middle-income countries, little is known about its contribution to the interruption of healthcare for people living with HIV in Ethiopia. In the 2020 report, the estimated number of PLHIV in Ethiopia was 616,105. Of these, 479,618 people living with HIV were receiving antiretroviral therapy (ART) (32). To improve healthcare delivery and provide sustainable quality care to PLHIV during the COVID-19 pandemic and future pandemics, we need a better understanding of the nature and scope of HIV treatment service interruption and its correlates. The study aimed to investigate the impact of COVID-19 on HIV healthcare services for PLHIV and identify the anticipated benefit of the COVID-19 vaccine in HIV services restoration in North Shewa, Oromia, Ethiopia. The results of this study will allow researchers and healthcare providers to understand and find solutions to reduce medical interruptions to PLHIV. In addition, it informs policymakers about planning and allocating resources for PLHIV in low- and middle-income countries and it can help to prepare for future public health emergencies.

## Materials and methods

### Study setting

**Figure 1:**
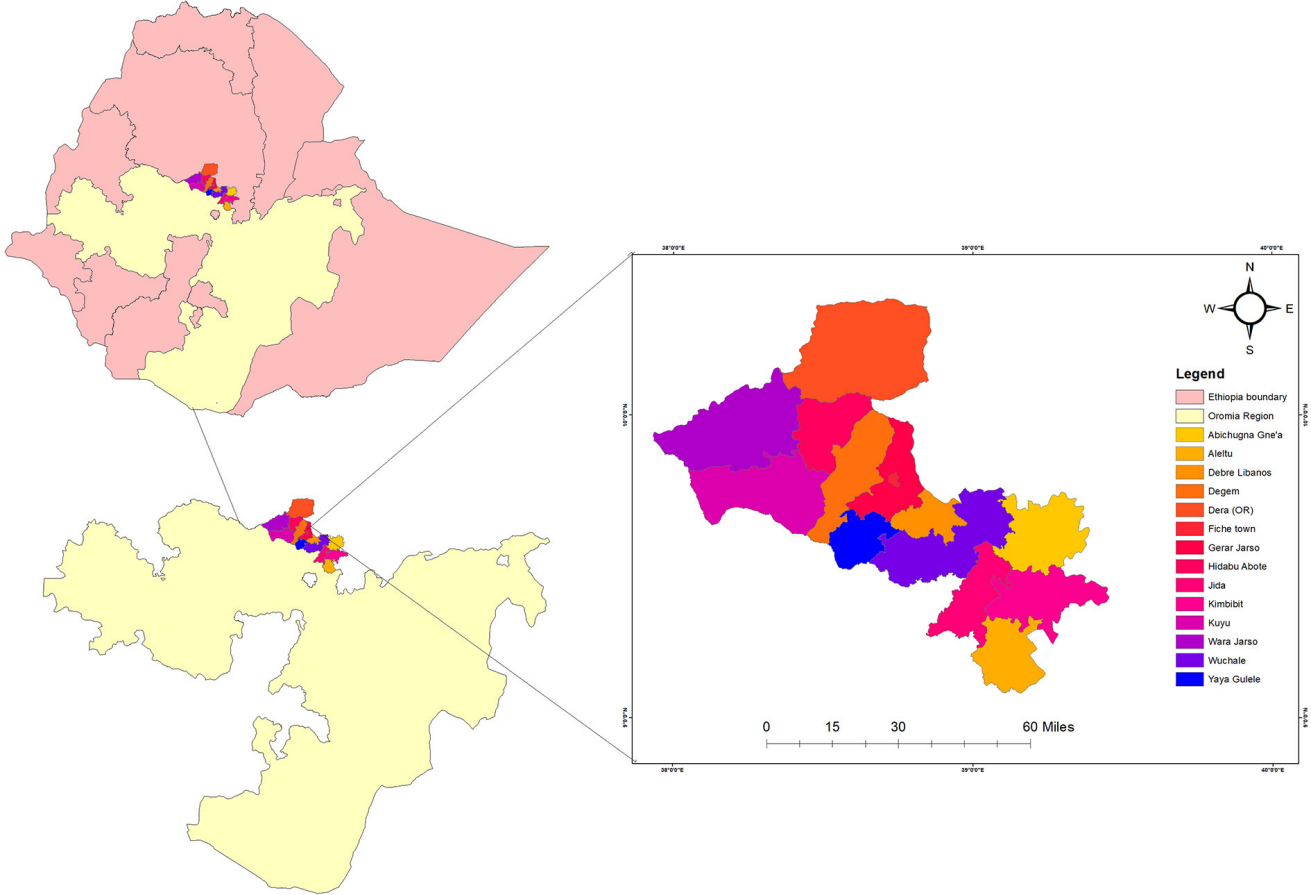
Map of North Shewa Zone, Oromia regional state, Ethiopia, 2021.

The study was conducted from June 2021 to July 2021 in all public hospitals and health centers in the North Shewa Zone of Oromia regional state in central Ethiopia, which provides health care for PLHIV. The zone is located in the northwest part of Ethiopia. According to the Central Statistical Agency of Ethiopia, the zone has a total population of about 1,639,586 of whom 717,552 and 922,034 are males and females respectively, with the majority of the population (89.75%) from rural residents. The study area has 13 administrative districts and one town administration (Figure 1). Likewise, according to the Zonal Health Bureau's 2019 annual report, the zone has five governmental hospitals and sixty-four health centers that provide service to the community. Among those, 13 Health Centers and 3 Hospitals are providing diagnostic and management care for PLHIV. During the study period, it was estimated that more than 5,430 PLHIV were receiving ART in North Shewa Zone.

### Study design

A qualitative method was used to explore how healthcare delivery is going on for HIV patients during the outbreak of COVID-19. To obtain details of the healthcare situation for PLHIV during the COVID-19 pandemic, in-depth interviews were adopted for this qualitative study. The face-to-face interview procedure in data collection allowed the participants to share their observations and experiences of providing healthcare services to PLHIV during the COVID-19 pandemic. The ART providers were given ample time to explain their ideas. This helped us to obtain a comprehensive picture of the participants' experiences and perspectives through multilevel themes. The study was reported based on the Combined Criteria for Reporting Qualitative Studies (COREQ) checklist (33).

### Study participants

Participants were selected from sixteen health facilities in the North Shewa Zone, Oromia region, Ethiopia. Participants who provided HIV care services in selected health facilities for more than 2 years were purposively selected to achieve our goals. On average, two ART providers were selected from each facility based on their experiences. Priority was given to ART providers with years of experience delivering HIV services. Based on these criteria, 32 ART providers were selected. Before the interview being conducted, all participants were informed of the purpose of this study, and written consent was obtained from each participant. Among thirty-two sampled participants, four were doctors, five were public health professionals, and twenty-three were nurses. All participants consented to participate in the interview. Although data saturation was reached around 16 interviews during our coding, we continued recruitment for convenience reasons and in case new possible themes emerged from subsequent interviews.

### Data collection

We conducted face-to-face semi-structured interviews to explore the participants' experiences with HIV care during COVID-19 and their expected impacts of COVID-19's vaccine on service restoration. To protect the privacy of participants, we provided codes instead of real names for all participants, and the interviews were conducted in a private setting one by one. Open-ended questions were drafted by the research team to probe into the detailed experiences of ART providers. The interviews were mainly focused on study objectives and probing questions were also used where necessary to get a more thorough understanding of the issue involved. We piloted the interview questions with other ART providers at other hospitals which were not included in this study. Based on the pilot interview, the interview outline was further edited and improved (see Additional file).

In-depth interviews were conducted to determine the impact of COVID-19 on HIV services and the expected benefits of COVID-19 vaccination in recovery services. Each interview lasted for approximately 45–60 min. The interviews were conducted in the local dialect (Afan Oromo). All interviews were audio-recorded. The principal investigator and the other two co-authors supervised all interviews and facilitated the interview process. Two public health professionals who can speak Afan Oromo fluently were selected for data collection based on their previous experience. Data collectors with experience trained in (study overview, objectives, participant selection, detailed tool review, interview approach, and role-play of interview skills). This method elicits candid responses in a private setting regarding professional topics of discussion. The interviewers have plenty of time to probe and obtain in-depth responses since respondents tend to express themselves more freely.

### Data management and analysis

**Figure 2:**
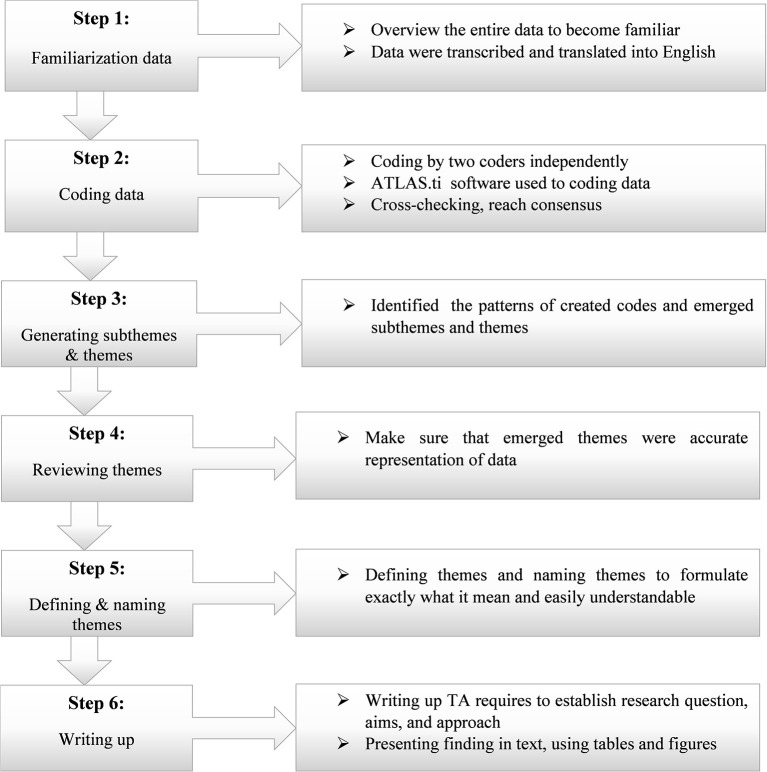
Illustrates the steps of thematic data analysis.

Data were analyzed using qualitative thematic analysis (TA) methods (34). The Consensus Qualitative Research (CQR) method was also used in conjunction with TA to study participants' experiences, attitudes, and beliefs. The conjunction of CQR and TA is used to reduce subjectivity in the coding process of research teams and increase the rigor of research methods (35). With this approach, subthemes, and themes were drawn from the data, providing the codes for the coding framework. Two experienced coders were involved in coding the data. First, the coders gave a comprehensive overview of the entire data to become familiar with its content. Before analyzing data, the interview data were transcribed and translated into English by a language expert. This means transcribing audio and organizing the field notes we took during the interview. Then the coders read the entire dataset to generate codes. Second, the translated data was copied to the ATLAS.ti version 7 for coding and analysis of the data (36, 37). Both coders coded data by using software independently. Then by using ATLAS.ti' inter-coder agreement (ICA), the discrepancies were checked and solved (38). Thirdly, based on the codes created, the coders identified the patterns among created codes and came up with subthemes and themes. Then their work was evaluated by the first author and discussed to achieve consensus before further analysis. Fourth, we make sure that our themes were accurate representations of the data by checking coding data with emerged themes. When certain codes were incorrect in a theme or emerging themes were not generic codes, we divided them up, combined them, discarded them or created new ones to make them more useful and accurate. Finally, we defined and named the themes and conducted the data analysis (Figure 2).

## Results

### Characteristics of study participants

Among participants (32 ART providers), most of the participants were men (25,). Participants were 26–55 years old, with an average of 33 years old (SD = 6.14). Participants work experience in providing ART ranges from 2 to 9 years, with an average of 4.53 (SD = 2.53) years. The disciplines of the participants were doctors, nurses, and public health officers, as shown in Table 1.

**Table 1:** Characteristics of study participants.

**Participants'** **characteristics**	**Distribution (*n =* 32)**
Gender	Male = 25, Female =7
Age	Mean = 33.72(SD=6.14)
Discipline	Doctor = 4, Nurse = 23, Public health officer = 5
Position	ART provider = 28, ART provider and ART physician = 4
Working experience on ART provider	Mean = 4.53(SD=2.53)
Health Facilities	From health centers = 26, From hospitals = 6
First dose COVID-19 vaccine status	Vaccinated = 31, Not vaccinated = 1

The findings of this study were discussed under three themes (interrupting HIV service delivery, innovative methods to HIV service delivery, and anticipated benefit of COVID-19 vaccine) and several sub-themes which emerged from the data analysis (Table 2).

**Table 2:** List of themes and sub-themes.

**Domain**	**Themes**	**Sub-themes**
HIV service delivery status	Interrupting HIV service delivery	Interruption of medical appointment
		Interruption of medicines supply
		HIV and HIV related tests interruption
		Lack of ART providers and PPE
	Innovative methods to deliver HIV service	Use telehealth to deliver HIV services
		Reducing frequency of visiting clinics
Demand for COVID-19 vaccine	Anticipated benefit of COVID-19 vaccine	Role of COVID-19 vaccine in HIV restoration services
		Role of COVID-19 vaccination in changing healthcare providers' attitude

### Interruption of HIV service delivery

#### Interruption of medical appointments

All participants reported that ART refilling and counseling appointments were disrupted during the COVID-19 outbreak. Before the COVID-19 outbreak, PLHIV visited ART clinics once every month to receive treatment and refill their ART, except for those on Appointment Space Model (ASM). Patients on ASM go to the ART clinic for treatment and refills ART once every 6 months. During the pandemic, to reduce the overcrowding of ART wards, the monthly visits of PLHIV to ART clinics were changed to once every 3 months.

Reducing the frequency of visits can appropriately reduce the use of ART regimens that affect ART adherence. Such disrupted appointments would have negative consequences, especially for PLHIV who were new to ART. Because they need frequent counseling and psychotherapy until they become stable. In addition, such patients require timely, supportive treatment and advice from physicians. However, because their medical appointments were reduced to every 3 or 6 months, they could not access vital services in time.

…*Changing the ART refilling time has a role in reducing adherence to ART. Because they may forget to use ART properly. (Participant 31, District 16 Hospital)*

…* In addition, for people who are just starting to receive ART, it is very difficult to stay for a long time without contact with healthcare providers. As you know, HIV can be very stressful for new patients until their condition is stable. (Participant 18, District 9 Hospital)*

Regarding the safety of ART regimens, some patients did not have appropriate medication storage to maintain their regimens and did not use medication as advised by their clinicians. This reduces the effectiveness of the ART regimen.

…*Schedule change may affect the safety of the drug. Some PLHIVs do not have proper medication storage. Especially new patients may not keep their drug in the proper place and may not use it appropriately. (Participant 02, District 1 Health center)*

#### Interruption of medicines supply

Most participants mentioned that, during the COVID-19 pandemic, healthcare facilities were unable to provide the usual HIV services as they did before the COVID-19 outbreak. Because of the lockdown and shutdowns of certain medicine manufacturers (39), opportunistic infection (OI) medicines and sometimes ART regimens were in short supply. In Ethiopia, ART and OI drugs are provided free of charge because of foreign aid. However, the delivery of OI medicines through aid has been discontinued due to the pandemic, so there were not enough OI medicines available to PLHIV for free. PLHIV had to pay for the OI medication. However, some PLHIV could not afford OI medicines.

*We have not provided all existing HIV services since the COVID-19 pandemic. We are facing a shortage of medicines. The supply of medicines is insufficient. Due to the lockdown, we are faced with a lack of ART and OI medicines. Most medicines, including ART medicines, are provided by foreign aid. The import system was blocked by the pandemic. Despite this, we still do not have enough OI medicines. This interrupted our provision of comprehensive treatment services. (Participant 02, District 1 Health center)*

…*Previously, opportunistic infections drugs were given for free to people living with HIV. But today, patients buy by themselves. This is difficult for patients who have no income and interrupted our service as well. (Participant 15, District 8 Health center)*

#### HIV and HIV-related tests interruption

HIV testing and management system were interrupted during the COVID-19 outbreak as mentioned by most of the participants. HIV testing is limited to hospitals and health centers, which means HIV testing is conducted at hospitals and health centers only. Services of outreach testing and family indexing testing were all stopped.

All participants stated that during the COVID-19 pandemic, all health facilities conducted HIV testing for the target group or key population (such as sexually transmitted infections (STI) patients, Tuberculosis (TB) patients, sex workers, drivers, pregnant women, gays, lesbians, etc.) among clients visiting health facilities. Some participants said that while voluntary HIV testing had taken place, the number of tests had dropped significantly. In addition, because of the lack of kits in some health facilities, counseling and HIV testing services were limited. Such interruptions of healthcare services may lead to a silently increasing prevalence of HIV in communities and increase the burden of the disease.

…* Since the onset of the COVID-19 pandemic, we are conducting HIV testing for targeted groups such as sexually transmitted infections patients, tuberculosis patients, sex workers, drivers, pregnant women, gays, lesbians, etc. In addition, we are testing volunteers. However, during the pandemic, the number of voluntary HIV testing has decreased significantly. The number of HIV tests during the epidemic is decreasing. This does not mean that the HIV prevalence rate has fallen. The prevalence of HIV in the community is quietly increasing as most people don't go to hospitals or other health facilities to get tested for HIV. If the outreach testing service starts, I am worried that the number of HIV-positive people will significantly increase. Currently, we do not perform outreach HIV testing. (Participant 19, District 10 Health center)*

…*Sometimes we faced a lack of kits, which interrupted HIV testing services in this health center. (Participant 14, District 9 Health center)*

In addition, during the COVID-19 pandemic, routine tests for PLHIV such as viral load testing and CD_4_ count were interrupted. Since all health centers did not have laboratory equipment to conduct these tests, the samples were sent to the nearby hospital for testing. However, due to restrictions on transportation and an increased number of discontinuation follow-ups, viral load tests, and CD_4_ count were reduced. This causes unable to know the viral level of PLHIV to provide healthcare accordingly.

…*Regular testing such as viral load testing and CD*_4_ *count is also interrupted due to the lockdown in sample transport. We have no laboratory materials to do those tests at this health center. Because of this, we are sending samples to other hospitals. During the emergent time, transport is locked and we faced difficulty sending samples. (Participant 02, District 1 Health center)*

#### Shortage of healthcare providers and personal protective equipment

As some participants reported, the COVID-19 pandemic caused a shortage of healthcare providers in certain medical institutions. Ethiopia, as a least developed country, lacked ART providers even before the COVID-19 outbreak. Due to the COVID-19 and lack of personal protective equipment (PPE), healthcare providers cannot provide services by protecting themselves. As a result, some healthcare providers were absent from medical institutions. The COVID-19 outbreak has exacerbated the shortage of healthcare providers.

…* Our country lacks healthcare providers. This is because the country's income is not enough to hire more healthcare providers, and Ethiopia does not have enough graduated health professionals. (Participant 02, District 1 Health center)*

*During the outbreak of COVID-19, we are facing a lack of ART providers, especially at the beginning. The number of ART providers in this hospital is tiny. During the COVID-19 outbreak, due to a lack of PPE, some ART providers were absent from work. Therefore, we are facing a shortage of ART providers. Workload has increased for the ART providers on duty. This situation is partially solved this year. But, as a hospital, we have no sufficient health care providers. (Participant 17, District 9 Hospital)*.

### Innovative methods to provide HIV services to PLHIV during the pandemic

As most participants described, contacting PLHIV during COVID-19 was very difficult. Due to the spread of COVID-19, PLHIV could not go to the ART ward as scheduled. This may cause poor adherence. Government and non-governmental organizations tried to reduce HIV service interruption by modifying the treatment and ART refilling schedule and using telehealth to contact PLHIV during the pandemic.

#### Use telehealth to deliver HIV services

Telehealth is an innovative technology that replaces face-to-face treatment in public health emergencies. This technology is highly used in developed countries (40) and many low and middle-income countries. In Ethiopia, some medical institutions supported by non-governmental organizations are experimenting with telehealth. Several participants mentioned they were contacting PLHIV through telehealth. However, the service has not been officially launched in Ethiopia.

*We are using telehealth services. This service was not officially started in Ethiopia. Some health institutions are supported by non-government organizations (NGO*_*s*_ *) to use telehealth to provide services for PLHIV. Our institution is one of the institutions that getting aid from NGOs for telehealth services. We are following the status of our clients and counseling by calling them. Especially during an emergency declared in our country, we are giving HIV services via telephone. (Participant 22, District 11 Health center)*

As described by most participants, if telehealth was formally implemented in Ethiopia, it would alleviate the burden of the healthcare system. Telehealth played a key role during the pandemic and other state emergencies. Because there is no direct physical contact between ART providers and PLHIV, the use of telehealth can reduce the risk of COVID-19 for both healthcare providers and PLHIV. It can protect the privacy of PLHIV, and reduce the cost and time of clinical visits. In addition, telehealth can reduce work overload at health facilities.

*For educated societies and developed countries, telehealth has many advantages. It can increase the attendance rate of patients. Save money spent on parking, transportation, child care, etc. Also, it can save time for work. For health care providers, it can reduce the work overload at the health centers. For developing countries, including Ethiopia, the telehealth advantage was outstanding. It had advantages for educated patients who have resources to use. It is used to keep the privacy of patients. Telehealth is very useful during an emergency time, especially for chronic patients, including PLHIV. (Participant 01, District 1 Health center)*

…* It can strengthen the relationship between PLHIV and healthcare providers. Also, it can reduce follow-up discontinuation as frequent contact with them. (Participant 12, District 6 Health center)*

Despite these advantages, telehealth has not been formally implemented in Ethiopia due to the lack of telephone, internet connectivity and other resources for some PLHIV. Also, lack of relevant education and misconceptions are barriers to the use of telehealth.

…* Telehealth has some disadvantages in Ethiopia. As Ethiopia is a developing country, its people do not have enough resources to use the advantages of technology. In addition, most PLHIVs are uneducated and can't use telehealth and telemedicine. Also, there are connection and network problems in some areas. There is no network access in some areas. Especially patients in rural areas are disadvantaged groups. According to my idea, the full implementation of telehealth services in Ethiopia will take time until society becomes familiar with this technology. (Participant 05, District 3 Health center)*

#### Reducing the frequency of visiting clinics

Many PLHIV discontinued follow-up and refilling ART because COVID-19 disrupted normal medical appointments for refilling ART and access to other HIV services. ART discontinuation can lead to poor ART adherence and drug resistance. In turn, it can lead to morbidity and mortality. To maintain their medical follow-up by preventing PLHIV from contracting COVID-19, it was necessary to improve the way HIV services were delivered. Based on this, the government decided to provide HIV services by reducing the frequency of visits to clinics to refill ART. Even though it was very difficult for some PLHIVs who need counseling and psychotherapy to stay without a doctor for a long time, there were advantages to keeping PLHIVs on treatment by preventing them from contracting COVID-19. With this approach, PLHIV could refill ART and other medications at least every 3 months.

*Before the COVID-19 outbreak, most PLHIVs refilled ART monthly and received counseling. During COVID-19, however, many PLHIVs stopped receiving ART on their appointments due to fear of contracting COVID-19. To reduce ART discontinuations, the government has modified ART refilling appointments. With this approach, infected people can receive counseling and supplemental antiretroviral therapy every three to six months, depending on their health status. (Participant 4, District 3 Health center)*

*Loss of HIV follow-up has increased since the outbreak of COVID-19. This leads to poor adherence to ART and the development of resistance. This condition contributes to HIV/AIDS morbidity and mortality. (Participant 03, District 2 Health center)*

### The anticipated benefit of COVID-19 vaccination in restoring HIV services

#### The role of COVID-19 vaccination in HIV restoration services

Most participants mentioned that the availability of the COVID-19 vaccine is changing the situation of healthcare systems all over the world. It can restore all HIV services. Healthcare providers can be present at health facilities and give full services without fear of the COVID-19 pandemic. After most residents are vaccinated, transport restrictions will be lifted and drug supplies will increase. ART and other drugs were imported from abroad via different aid channels. Such import systems have been interrupted during the COVID-19 pandemic. In addition, regular testing services such as viral load tests and CD_4_ count will be restored as transportation restrictions are lifted. Outreach HIV testing will start and help to reduce the transmission of HIV.

*If most people are vaccinated, normal health care services can resume. Transport restrictions will lift and we can get drug supply from abroad since ART and most OI drugs are imported from abroad. It can reduce losses and drop. Regular testing services such as viral load tests and CD*_4_ *count will start. Also, outreach HIV testing will start, which would help to reduce the silent transmission of HIV. (Participant 11, District 6 Health center)*

…* This means, that all healthcare providers can be available and give full services for PLHIV. Also, PLHIV can attend according to their follow-up schedule to refill ART…(Participant 09, District 5 Health center)*

However, some participants doubt the effectiveness of the COVID-19 vaccine for different variants of COVID-19. As the coronavirus has mutated to become more contagious over time, it is difficult to say the COVID-19 vaccine can restore all HIV services until its effectiveness has been proven in all types of COVID-19 variables.

*I don't think the vaccine will impact the restoration of services because COVID-19 changes its structure every time. However, if these vaccines are effective against all types of COVID-19, they can restore normal HIV services...(Participant 05, District 3 Health center)*

… *I am concerned about the effectiveness of vaccines against delta COVID-19. Since COVID-19 can mutate, the effectiveness of the vaccine is questionable to me. I recommend that people use preventive materials as much as possible....(Participant 04, District 2 Health center)*

#### The role of COVID-19 vaccination in changing healthcare providers' attitude

Most participants have a positive attitude about the COVID-19 vaccine being developed. They believe a COVID-19 vaccine could reduce the spread of COVID-19. Even they believe that if most people are vaccinated and protective materials are used appropriately, COVID-19 will be over in the short term. The vaccine increased their self-confidence, and they started full services for PLHIV after vaccination. They said they feared COVID-19 to provide comprehensive HIV services for PLHIV until they were vaccinated. That changed with the development of a COVID-19 vaccine.

*With the outbreak of COVID-19, I am very depressed. I sometimes miss work because of the fear of COVID-19. Because healthcare providers are more vulnerable to COVID-19 as they are on the front lines fighting this pandemic. We do not provide appropriate services for PLHIV. This situation may increase the country's disease burden. However, after the development of a COVID-19 vaccine, this difficult situation is gradually diminishing. I got my first dose of the COVID-19 vaccine. After I am fully vaccinated, I was no longer afraid of contracting COVID-19. Vaccines give me the confidence to provide a full service. (Participant 13, District 7 Health center)*

However, as most vaccinated people stopped using protective materials, some participants were concerned about the spread of COVID-19. Since a vaccine cannot 100% stop the spread of COVID-19, it can cause other waves of COVID-19.

*The presence of the COVID-19 vaccine may lead HIV patients and healthcare providers to reduce to use of COVID-19 prevention materials. Some people think they will never get COVID-19 after being vaccinated, which is the wrong attitude. (Participant 11, District 6 Health center)*

*Starting with health professionals, vaccinated people stopped using protective materials such as masks. This may increase the spread of COVID-19 again because the vaccine is not 100% prevent. It is not the time for complacency or carelessness. I recommend that everyone who has been vaccinated and who has not been vaccinated, use protective materials to prevent the spread of COVID-19. (Participant 18, District 9 Hospital)*

## Discussion

This study evaluates the impact of the COVID-19 pandemic on HIV services and the anticipated benefit of vaccination on the restoration of HIV services. ART providers were given ample time and opportunity in this study to share their feelings, perspectives, and experiences on this issue. Through this research, we found the following problems. First, we found that HIV services were disrupted by the COVID-19 pandemic. These disruptions were medical appointments, service coverage, and lack of ART providers. The timing of refills of ART was changed from monthly to every 3 months. PLHIV received counseling services during their visit to the clinic for refilling ART. However, the new appointment policy did not take into account the patient's lifestyle. ART and other medications require proper storage. Some patients, especially those new to ART and those living in rural areas, do not have suitable storage. This can reduce the effectiveness of the drug. New patients require counseling each time until they are acquainted with the medication regimen. New HIV-positive patients should be closely followed up to check for adverse drug reactions.

In addition to disruptions to medical appointments, most health facilities could not provide previous services during the pandemic. Healthcare providers did not have enough PPE to provide comprehensive and cost-effective healthcare. This puts healthcare providers and clients at risk of COVID-19 (41, 42). Due to the lockdown, some healthcare facilities were facing shortages of medicines such as ART and OI drugs. Besides, routine tests, such as viral load tests and CD4 counts, were discontinued in the first year of the pandemic. With such tests, the follow-up status of the patient can be determined. However, without these tests, it's difficult to know the health outcomes of PLHIV. Loss-to-follow-up during the pandemic were increasing due to a lack of HIV services and fear of the spread of COVID-19 (43). Follow-up discontinuation from ART is the major contributor to attrition and further poor quality of life and death (44, 45). Also, the subsequent withdrawal of PLHIV impacted ART adherence. Good ART adherence is associated with greater viral suppression (46), while low or poor ART adherence can lead to poor mental and physical health outcomes (47).

We observed a shortage of healthcare providers in some facilities during COVID-19. Without enough PPE, healthcare providers were afraid to provide services during COVID-19. We found that, in Ethiopia, there is a lack of ART providers, which may be related to national income, and the inability to hire enough health sector professionals.

Second, the study identified innovative ways to deliver HIV services during the COVID-19 pandemic. These were using telehealth technology and reducing the frequency of visits to medical facilities. These approaches helped to deliver HIV services by preventing PLHIV from contracting the COVID-19 pandemic. Especially telehealth technology was used during emergency times like COVID-19 (22, 40, 48–50). Various studies have reported the use of telehealth technology to replace medical and non-medical treatment in medical institutions (20–25). Ethiopia has not officially launched telehealth services. However, we have found that if it starts in Ethiopia, it has many advantages, especially during public health emergencies. It was used to continue the provision of health care for PLHIV (28) and reduce overcrowding in medical facilities. This helped us reduce the spread of COVID-19. Although these innovative approaches are used to prevent PLHIV from contracting COVID-19, it has a negative impact on the proper delivery of HIV care, especially for PLHIV who newly started ART and PLHIV with unstable health status. PLHIV, newly exposed to ART and PLHIV with unstable health status, requires close follow-up until stable. Although further research is needed, for such patients, reduced frequency of visits time may have adverse consequences, such as inappropriate medication and drug resistance.

Third, we found that interruption of HIV testing, HIV-related tests, and a shortage of HIV testing kits in some health facilities during COVID19. during COVID-19. During the pandemic, HIV testing and counseling services have been limited to healthcare facilities. HIV testing was conducted only for the target population among the clients of the medical facility. Before the outbreak, unrestricted voluntary HIV testing and outreach HIV testing services were provided in highly-populated places such as schools and markets. However, due to the spread of COVID-19, such services were interrupted. To prevent the spread of the coronavirus, the number of patients visiting medical facilities has been reduced (17, 30, 31). In addition, the shortage of HIV testing kits in some health facilities is directly related to the decline in HIV testing services. Reducing HIV testing may increase HIV prevalence in the community. Viral load testing and CD_4_ counts have been disrupted during COVID-19. Both tests show us the status of PLHIV and the effectiveness of ART treatment (51). However, due to the pandemic, these important tests have been disrupted, and there is no way to know the effectiveness of the ART regimen.

Fourth, we identified the anticipated benefits of a COVID-19 vaccine in restoring HIV services. A COVID-19 vaccine could restore normal health care by limiting the spread of the COVID-19 pandemic (52). If most of the world were vaccinated against COVID-19, transport between countries would begin as it was before the outbreak. This has helped us get adequate supplies of medicines, which have been reduced due to the lockdown. Additionally, vaccinated healthcare providers can provide services without fear of COVID-19. However, as different variants of COVID-19 develop from time to time, the effectiveness of the COVID-19 vaccine is in doubt. This situation could reduce confidence in the effectiveness of a COVID-19 vaccine. Today, hesitancy to vaccination was recognized by the world health organization as one of the most important health threats in the world (53). This condition was also reported for the COVID-19 vaccine. Without considering the risks of the COVID-19 pandemic, a significant portion of the world population is reluctant about the COVID-19 vaccine (54). To change this skepticism, healthcare professionals and governments are working to raise awareness of the importance of vaccines, compared to the risks of the COVID-19 pandemic. People trust healthcare professionals and science to foster confidence in vaccines.

Finally, based on participants' experiences, we identified gaps in the proper delivery of HIV care during the COVID-19 pandemic in Ethiopia and suggest possible ways to prepare for future public health emergencies. We found gaps to prepare for public health emergencies. The government pays less attention to the health sector. Therefore, health departments were not as prepared as possible for public health emergencies. As COVID-19 entered Ethiopia, the government did not have enough materials (e.g., PPE, COVID-19 testing machines, etc.) to prevent its spread. Samples have been sent to other countries to test for COVID-19. These gaps stem from a lack of prior preparation for public health emergencies. Hence, governments learn from COVID-19 to prepare for future endemic or pandemics. The study suggests that the Ethiopian government should assemble sufficient manpower to learn the latest skills, train emergency response teams, and prepare an independent emergency budget.

## Conclusion

This study identifies the overall impact of COVID-19 on HIV services in Ethiopia. The HIV service schedule has changed to reduce the spread of COVID-19 but impacts the quality of HIV care. Health facilities could not provide usual HIV services during the COVID-19 pandemic. This may lead to increase subsequent discontinuation, resulting in poor adherence and low rates of viral suppression. As previous studies indicated, disruption of ART could lead to extra deaths from AIDS-related illnesses in sub-Saharan Africa in 2020–2021 (11). Also, this study identifies the importance of a COVID-19 vaccine in restoring HIV services. COVID-19 vaccines help slow down the spread of COVID-19 and help medical facilities function properly. This research guides governments to identify their gaps and prepare for future public health emergencies.

## Data availability statement

The original contributions presented in the study are included in the article/supplementary material, further inquiries can be directed to the corresponding authors.

## Ethics statement

The study was approved by the research Ethics Committee of the School of Public Health, Xi'an Jiaotong University (Ref No. 2021-1482) and North Shewa Zone Health Bureau, Oromia regional state, Ethiopia (Ref No. WEF/4308/6/35). The letter was sent to all concerned bodies and permission was obtained from respective health facilities. Informed consent was obtained from all study participants.

## Author contributions

AA is the principal investigator of the study and conceived and planned the study. AA, GG, and TG were involved in data collection, extracted data, analysis, and drafting of the manuscript. LZ, RZ, JS, WZ, and MZ made a substantial revision of the manuscript. LZ and RZ supervised the study. All authors gave final approval for the work to be published.

## Funding

This study was supported by the Bill & Melinda Gates Foundation (Grant Number: INV-006104), the National Natural Science Foundation of China (Grant Number: 81950410639), Outstanding Young Scholars Support Program (Grant Number: 3111500001), Xi'an Jiaotong University Basic Research and Profession Grant (Grant Numbers: xtr022019003 and xzy032020032), Epidemiology modeling and risk assessment (Grant Number: 20200344), and Xi'an Jiaotong University Young Scholar Support Grant (Grant Number: YX6J004).

## Conflict of interest

The authors declare that the research was conducted in the absence of any commercial or financial relationships that could be construed as a potential conflict of interest.

## Publisher's note

All claims expressed in this article are solely those of the authors and do not necessarily represent those of their affiliated organizations, or those of the publisher, the editors and the reviewers. Any product that may be evaluated in this article, or claim that may be made by its manufacturer, is not guaranteed or endorsed by the publisher.

## Supplementary material

Supplementary Table 1 Interview protocol table.
