# Supplementary material for: Impact of COVID-19 on HIV services and anticipated benefits of vaccination in restoring HIV services in Ethiopia: A qualitative assessment
Source: Front Public Health. 2022 Nov 3;10:1033351. doi: 10.3389/fpubh.2022.1033351 (PMC9671075; doi:10.3389/fpubh.2022.1033351)
Supplement: Supplementary Table 1 — Interview protocol table. [file Table_1.DOCX]

Interview protocol table

| **Part I: Background information**  Name of health facility____________________  Discipline/profession ________________  Position ________________  Age of participant ___________  Sex of participant___________  When were you employed in this facility ___________?  For how long have you worked in the HIV care unit/this position______________?  **Part II: Interview Guide**   1. During the COVID-19 pandemic, does this medical facility provide HIV care as usual? If not, please explain how it works!   (Probe: What are some of the main things that make it difficult to give appropriate care for PLHIV during COVID-19?)  1.1. Before the COVID-19 outbreak, how often did your institution distribute ART?  1.2. During the COVID-19 outbreak, how often does your institution distribute ART?  1.3. How to detect and manage HIV during the COVID-19 outbreak?  (Probe: Explain whether there were differences in HIV testing, viral load testing, and CD_4_ T-cell counts during the outbreak by comparing pre-outbreak conditions.)   1. Do you think most HIV healthcare providers’ available during the outbreak of COVID-19?   (Probe: Did this shortage happen before the COVID-19 outbreak?   1. How do you deliver HIV services during COVID-19 pandemic?    1. Is this health facility use telehealth to deliver service for PLHIV during the outbreak of COVID-19?    2. How do you explain the advantages and disadvantages aspects of telehealth in Ethiopia? 2. What are expected benefits of COVID-19 vaccine on HIV restoration service and health care providers’ attitude?   (Probe: Are you vaccinated? How many doses did you get?   1. What do you recommend for a future public health emergency to improve early detection and management?   Thank you for your participation in this interview. We appreciate the time you took to talk with us. Do you have any questions about the interview or the study that I can answer?  How was the interview experience for you? Was there anything that made you uncomfortable or feels offended in any way?  Is there anything you would like to tell me that was not covered in the interview? Are there any questions that you would like me to ask in future interviews, or is there anything that you would want to know about regarding this topic – what we have discussed? |
| --- |
